# Gene Regulation Using Spherical Nucleic Acids to Treat Skin Disorders

**DOI:** 10.3390/ph13110360

**Published:** 2020-11-02

**Authors:** Thomas R. Holmes, Amy S. Paller

**Affiliations:** Department of Dermatology, Northwestern University Feinberg School of Medicine, Chicago, IL 60611, USA; thomas.holmes@northwestern.edu

**Keywords:** nanoparticles, spherical nucleic acids, gene therapy, psoriasis, diabetes, wound healing, skin cancer

## Abstract

Spherical nucleic acids (SNAs) are nanostructures consisting of nucleic acids in a spherical configuration, often around a nanoparticle core. SNAs are advantageous as gene-regulating agents compared to conventional gene therapy owing to their low toxicity, enhanced stability, uptake by virtually any cell, and ability to penetrate the epidermal barrier. In this review we: (i) describe the production, structure and properties of SNAs; (ii) detail the mechanism of SNA uptake in keratinocytes, regulated by scavenger receptors; and (iii) report how SNAs have been topically applied and intralesionally injected for skin disorders. Specialized SNAs called nanoflares can be topically applied for gene-based diagnosis (scar vs. normal tissue). Topical SNAs directed against TNFα and interleukin-17A receptor reversed psoriasis-like disease in mouse models and have been tested in Phase 1 human trials. Furthermore, SNAs targeting ganglioside GM3 synthase accelerate wound healing in diabetic mouse models. Most recently, SNAs targeting toll-like receptor 9 are being used in Phase 2 human trials via intratumoral injection to induce immune responses in Merkel cell and cutaneous squamous cell carcinoma. Overall, SNAs are a valuable tool in bench-top and clinical research, and their advantageous properties, including penetration into the epidermis after topical delivery, provide new opportunities for targeted therapies.

## 1. Introduction

Gene modulation as a therapeutic approach has become increasingly viable, given the progress made in developing efficient, non-toxic systems for the delivery of gene targeting oligonucleotides. One such technology, which has already moved to clinical trials, is the spherical nucleic acid (SNA). SNAs are spherically-oriented nanostructures with duplexed or single-stranded oligonucleotides, often anchored to a central nanoparticle. The spherical structure of these nanoparticles imparts SNAs with enhanced nucleotide stability and improved cellular uptake, making them useful tools for gene modulation both in vitro and in vivo. A unique feature of SNAs is their ability to penetrate the epidermal barrier and into the dermis, significantly suppressing expression of targets in intact skin. As such, SNAs have moved forward towards topical and intralesional therapy for psoriasis, diabetic wound healing, scars, and skin cancer.

## 2. Introduction to SNAs: Structure and Synthesis

### 2.1. SNA Structure

#### 2.1.1. SNA Core

Spherical nucleic acids are a class of gene modulatory molecules that share the spherical structure of densely oriented nucleic acids anchored to a central core. First described in 1996, the original SNAs were 60–80 thiol-conjugated DNA oligomers in a spherical orientation around a 13 nanometer (nm) gold nanoparticle core (originally called DNA Au-NPs) [1]. Since their initial creation with Au-NP cores, a wide variety of inorganic (silver, iron oxide, quantum dots, or silica) or organic materials (proteins, polymers, DNA, or liposomes) have been developed to serve as the SNA core [2,3,4,5,6]. Depending on the type of core used, magnetic, luminescent, electrical, catalytic, light scattering, and spectroscopic properties can be conferred upon SNAs for use as molecular probes or assembly into complex structures [7,8]. For example, magnetic and conductive properties of SNA cores allow them to be used in a wide range of microarray-based assays for detection of specific oligonucleotides or proteins [8,9,10,11,12,13,14]. Coreless SNAs have also been produced as self-assembling, spherically-oriented oligomers or, alternatively, by creating Au-NP SNAs, catalytic crosslinking of the functionalized oligo at the base of the strand closest to the core, and then dissolving the gold core using potassium cyanide, leaving behind a hollow SNA [15,16]. These coreless nanoparticles eliminate the potential long-term toxicity of the core (albeit low-risk) and highlight that the chemical and physical properties of SNAs, including their facile cellular uptake, are governed by the oligonucleotide conjugates themselves, rather than the core [17,18]. Au-NPs were originally chosen for the SNA core material because they are easy to synthesize, can be made highly monodisperse, can be conjugated to many types of ligands, have well-defined catalytic properties, and are readily tracked by inductively coupled mass spectrometry, all of which makes them a popular choice for SNA use in vitro.

#### 2.1.2. Oligonucleotide Attachment to SNA Core

The oligonucleotides used for SNAs typically have three main components: (i) a terminal moiety that aids in its attachment to the core; (ii) a spacer region; and (iii) a specific functional region that is designed for target gene recognition. In the case of gold core SNAs, the oligonucleotide is commonly functionalized with a thiol group (termed alkylthiol-DNA/RNA) on one terminal end, facilitating the attachment of alkylthiol-DNA/RNA to the core surface via gold-sulfur bonds [1]. However, modification of the thiol moiety can enhance the oligo Au-NP interactions, including the use of cyclic disulfides or branched thiol groups [19,20]. When creating SNAs using silver nanoparticle cores (Ag-NPs), cyclic disulfides are preferable due to the more unstable surface of Ag-NPs [21]. Thiol-modified oligonucleotides are also used when making certain coreless, silica, or quantum dot core SNAs, in which Au-NPs are used as a scaffold [5,15,22]. Self-assembling or protein core SNA oligos contain a dibenzocyclooctone-amine (DBCO) moiety which facilitates its attachment to azide groups on polymers using click chemistry [16,23,24]. When generating liposomal SNAs, a tocopherol moiety is used for attachment to the liposomal core [5].

#### 2.1.3. Oligonucleotide Spacer Region

Construction of the spacer region is also important to maximize SNA stability and oligonucleotide load. The spacer gives the conjugated oligonucleotide more flexibility and provides a buffer from the cationic core, which allows for more efficient hybridization with its target sequence. Spacers consisting of a 10 thymine/alanine sequence or polyethylene glycol (PEG) are commonly used due to their low interaction with the metal cores, thus increasing DNA/RNA load [25]. A polythymine spacer sequence has been shown to protect SNAs from salt-induced aggregation [26]. SNA oligos used for coreless SNAs contain modified nucleotides in the spacer region with an alkyne group, which allows for the catalytic crosslinking of nucleotide strands [15]. Another crosslinking method utilizes a thymine spacer with C6-amines which can be crosslinked with PEGylated bis(sulfosuccinimidyl)suberate [22].

#### 2.1.4. Oligonucleotide Targeting Sequence

The conjugated oligonucleotide sequence that targets the gene of interest is located on the end farthest from where the oligonucleotide attaches to the nanoparticle core. Rosi et al. were the first to show that SNAs could enter a cell and knockdown gene expression using single-stranded DNA to target GFP expression [27]. Since then, duplexed DNA, siRNA or miRNA conjugates have been attached to SNA cores to regulate gene expression [18,28,29,30,31]. Catalytic RNAs, termed ribozymes, have been loaded onto SNAs to directly cleave the target mRNA strand as opposed to relying on the native RNA interference (RNAi) pathway for cleavage [32]. DNA can be loaded onto SNA cores at about 60–80 strands per core, whereas RNA has been loaded onto cores at about 30–45 RNA strands per core [29,30]. The inherent instability of RNA compared to DNA is a factor to consider when designing SNAs for gene regulation. To achieve similar stability to DNA-SNAs, RNA-SNAs can be passivated with PEG, which risks reducing cellular uptake, or the RNA is synthesized using modified nucleotides (i.e., 2′-O-methyl) to increase stability [33,34]. Phosphorothioate oligonucleotides, which replace the non-bridging oxygen in the natural phosphodiester bond of DNA/RNA backbones with a sulfur bond, increase oligomer stability and have been shown to promote their nuclear retention, allowing one to specifically target nuclear sequences, for example long non-coding RNAs [35]. Despite the challenges posed by RNA instability in SNA production, RNA-SNAs are still easily taken up by cells, have improved stability (when compared to linear RNA) through functionalization of RNA onto the solid core, can utilize the intracellular RNAi pathway machinery, and allow for more precise targeting, including with single base pair accuracy [28].

#### 2.1.5. Oligonucleotide Functional Additives

Oligonucleotide conjugates can be functionalized with other groups to tailor their utilization. For example, fluorescent tags can be added to track cellular uptake or detect unique gene expression through attaching specialized “nanoflares”, dyes attached to detect cellular levels of analytes, or antibodies can be complexed with SNAs to detect proteins [6,12,25,36,37,38]. Whereas most SNAs aim to knock down expression of a specific gene of interest, nanoflares utilize a short, mRNA-targeting DNA sequence attached to a fluorophore that is quenched due to its close proximity to the nanoparticle surface to allow detection [39]. Upon binding to the mRNA target and dissolution from the nanoparticle, the fluorophore is no longer quenched and thus produces a signal [39]. A similar SNA technology, “sticky-flares”, uses longer antisense sequences that allow the fluorophore to remain bound to the transcript, enabling the tracking of mRNA movement in the cell and changes in gene expression in real time [40]. Rapidly proliferating cancer cells with high telomerase activity can be identified using SNA oligos functionalized with telomerase primers, while immune cells can be conditioned to target cancer cells by loading SNAs with cancer cell lysates or antigens [41,42,43]. Antibodies can be conjugated to the DNA or absorbed onto the SNA for cell-specific targeting [44]. SNAs have also been used to increase contrast in magnetic resonance imaging (MRI) to enhance uptake of Gd (III) chelates into cells [45]. Owing to their favorable properties of rapid cell uptake and stability, SNAs have also been used for drug delivery by loading chemotherapeutic agents onto the SNA shell or into the SNA core [6,46,47].

### 2.2. SNA Synthesis Using an Au-NP Core

**Figure 1:**
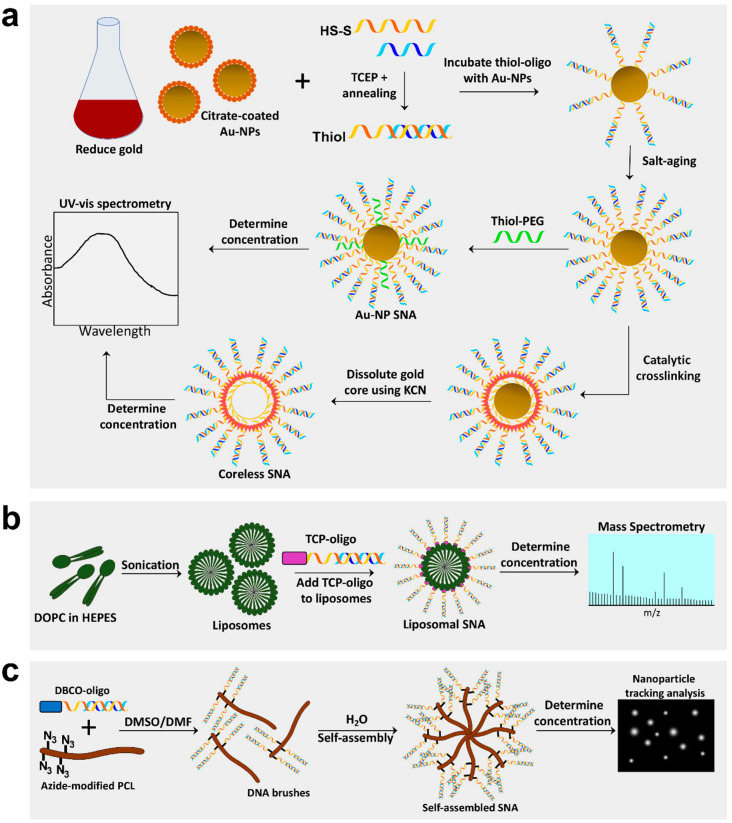
Synthesis of Au-NP core, coreless, liposomal core, and self-assembling spherical nucleic acids (SNAs). (**a**) Gold nanoparticle (Au-NP) SNAs are produced by reducing gold into 13 nm Au-NPs and preparing oligos by annealing sense and antisense strands and cleaving the disulfide bond to create a free thiol group. Au-NPs are incubated with thiol-oligos, salt-aged to increased oligo density, and thiol-polyethylene glycol (PEG) added to fill empty spaces. Lastly, Au-NP SNA concentration can be determined by spectrophotometry. Coreless SNAs are produced by crosslinking oligo strands on the surface of the Au-NP, then dissolving the gold core using potassium cyanide (KCN). (**b**) Liposomal SNAs are synthesized by sonicating 1,2-dioleoyl-sn-glycero-3-phosphocholine (DOPC) free fatty acids in HEPES buffer to create liposomes ~30 nm in diameter; liposomes are incubated with oligos containing a tocopherol (TCP) moiety for integration into liposomes. L-SNA concentration is determined by mass spectrometry. (**c**) Self-assembling SNAs are produced by conjugating oligos with a dibenzocyclooctone-amine (DBCO) moiety to polycaprolactone (PCL) polymers modified with azide groups in a DMSO/DMF solution to create DNA brush polymers. The DNA brushes then self-assemble in the presence of water and the concentration can be determined using nanoparticle tracking analysis.

Synthesis of SNAs is often initiated by first producing the core material. Au-NPs are a common core used for SNA production and in many cases act as a scaffold when synthesizing silica-core or coreless SNAs [5,15]. Au-NPs are typically synthesized by reducing the gold (III) chloride dihydrate through boiling in the presence of sodium citrate [48] (Figure 1a). This method of gold reduction, called the Frens method, produces gold particles in the range of 12–64 nm, depending on the molar ratio of citrate to gold [48]. The solution turns from yellow to gray to eventually a deep red, which indicates that the gold particles have been reduced [1]. There are several methods for Au-NP reduction that can produce particles of various sizes (reviewed in [49]). The smaller the diameter of the Au-NP core, the greater the density of oligonucleotide that can be loaded onto the Au-NP; however, the larger the nanoparticle, the more total oligomers that can be loaded (for example, one can load up to 600 strands on a 30 nm particle). This phenomenon is explained by the increased radial curvature of the smaller Au-NP, which increases the deflection angle between DNA, thus decreasing steric hindrance and electrostatic repulsion between strands [50]. The final concentration of Au-NPs is determined by measuring the absorbance (A) on a UV-visible spectrophotometer (for example, at a wavelength of ~520 nm for the gold nanoparticles) [25]. The concentration of Au-NP SNAs can then be determined using Beer’s Law (A = εLc) where A is the absorbance measured, ε is the extinction coefficient, which is based on the Au-NP diameter, and L is the path length (typically 1 cm) [22].

After reduction, the citrate-coated Au-NPs are treated with diethyl pyrocarbonate and autoclaved to inhibit nucleotide degradation and sterilize the particles, respectively. This step is especially critical for the production of RNA-conjugated SNAs. Before adding the oligonucleotide to the Au-NPs, the oligo strands may need to be prepped for conjugation to the Au-NP core. For example, thiolated oligos (like those from Integrated DNA Technologies) are capped with a disulfide bond, so it must be reduced using tris(2-carboxyethyl)phosphine (TCEP) to expose a free thiol for attachment to the core. Additionally, if using duplexed thiol-DNA or RNA, a duplex formation reaction is performed to anneal the sense and antisense strands (single-stranded thiol-DNA or RNA skips this step). Once ready for Au-NP conjugation, single-stranded or hybridized thiol-oligonucleotide strands are added to the Au-NP solution at a ratio of ~20:1 (oligo:Au-NP) and incubated at room temp for about one hour (Figure 1a). Next, sodium salt is added to the DNA/RNA Au-NPs at a concentration of 50–150 mM, incubated overnight, and then slowly increased in 50 mM increments until the final concentration reaches 350 mM, a method called “salt-aging” [25,51] (Figure 1a). The negatively charged DNA repels the insertion of more DNA duplexes, so the addition of the positively charged sodium ions blocks these repulsions, allowing higher densities of DNA on the Au-NP core [51]. It is critical that salt-aging is performed gradually during a period of 1–2 days to prevent aggregation of SNAs. However, salt-aging can be hastened considerably by reducing the reaction pH to 3.0. Pre-treatment of Au-NPs with a surfactant (for example, Tween 20), reduces Au-NP aggregation during salt-aging and facilitates oligonucleotide attachment to the core [52]. The high DNA density and strong sulfur-gold interactions (which displaces the much weaker citrate-gold interactions) give Au-NP SNAs a negative zeta potential (<−30 mV), which ultimately helps prevent their aggregation [53]. After oligo conjugation to the Au-NP core, coreless SNAs can be produced by catalytic crosslinking of the oligos via the modified nucleotides in the spacer region, and then dissolving the gold core using potassium cyanide (Figure 1a).

Once the Au-NPs are fully saturated with oligonucleotides, any empty spaces remaining on the surface of the SNA core are backfilled with thiol-conjugated PEG to further improve stability. Bromide has also been used as a backfiller that can efficiently displace improperly attached oligos on the Au-NP surface [54]. The SNAs are concentrated using centrifugal filtration units and washed several times with 1× phosphate buffered saline to remove residual surfactants, salts, or unattached DNA/RNA which could inhibit downstream applications. The final concentration of Au-NP SNAs is determined by measuring the absorbance (A) on a UV-visible spectrophotometer and Beer’s Law (A = εLc) as described above. To determine the oligo concentration, the nucleotide strands can be dissociated from the gold core with dithiothreitol treatment, Au-NPs removed by centrifugation, and then absorbance measured on a UV-visible spectrophotometer at 260 nm. The concentrated SNAs can be used directly on cells in culture (both 2D and 3D) or topically in vivo, such as on mouse skin. As described above, SNA’s enhanced stability allows oligo SNAs to remain intact for months both at room and physiological temperatures.

The requirement for thiol conjugation of siRNAs to SNA cores for each targeting siRNA translates into highly expensive, specially ordered oligonucleotides. One approach to this problem is the use of universal SNAs that contain DNA anchors and a generated sense strand of siRNA that can be ligated to the DNA [55]. This also saves time, as new siRNA-SNAs can be produced through simple ligation and annealing reactions instead of full SNA production for each target.

### 2.3. Generation of Liposomal SNAs

Liposomal SNAs (L-SNA) are a newer SNA class using ~30 nm diameter unilamellar vesicles as the SNA core, prepared by sonication of 1,2-dioleoyl-sn-glycero-3-phosphocholine (DOPC) in HEPES buffered saline [2] (Figure 1b). Oligonucleotides are attached to L-SNA cores using a tocopherol (TCP) moiety on the terminal end, which is absorbed by the liposomal core via hydrophobic interactions [2]. The TCP-oligos are incubated with liposomal cores at a ratio of 100:1 oligo to liposomes and the formed L-SNAs are then concentrated using tangential flow filtration with a 30 kD weight cutoff. Similarly to Au-NP SNAs, the oligo concentration can be determined by dissolving the liposomes in 90% methanol to dissociate the oligonucleotides from the core, and then absorbance is measured on a UV-visible spectrophotometer at 260 nm. The concentration of L-SNA liposomal cores can be determined using mass spectrometry (see supplementary methods in [5]).

Zhang et al. has created liposomal-based SNAs using biodegradable DNA-brush block copolymers (DBBC) [16]. Multiple oligonucleotides share a hydrophobic lipid tail, which can self-assemble into a micelle liposomal core. DBBC-based L-SNAs display increased oligonucleotide density (and thus a greater negative charge), higher melting temperature, and greater cellular uptake compared to linear DNA block copolymer L-SNAs [16]. DBBC-based L-SNAs are produced by modifying polymers with azide groups which allows for the attachment of DBCO-oligos in the presence of an organic solvent (i.e., DMSO, DMF) using click chemistry (Figure 1c) [16]. Replacing the organic solvent with water by dialysis initiates self-assembly of the DBBC polymers into a micelle-like SNA roughly 40 nm in diameter [16]. Unlike Au-NP SNAs, these self-assembled micelle SNAs cannot be quantified by spectrophotometry and thus must be measured using nanoparticle tracking analysis (Figure 1c) [56].

Modification of the oligonucleotide attachment moiety can impact the activity and distribution of L-SNAs. Attaching DNA sequences to L-SNAs using a low-affinity cholesterol tail (CHOL-LSNA) or high-affinity diacylglycerol lipid tail (DPPE-LSNA) reduces the inflammatory cytokine response in mice after intravenous injection compared to free DNA [57]. In vitro, CHOL-LSNAs are less readily taken up by cells compared to DPPE-LSNAs [58]. When CHOL- and DPPE-LSNAs were functionalized with the same immunostimulatory oligonucleotides, DPPE-LSNAs were more potent stimulators of macrophages [58]. Similar to coreless SNAs, use of L-SNAs avoids the possible long-term toxicity issues that accompany use of metal core SNAs. For this reason, L-SNAs are the preferred SNA subtype for preclinical and clinical studies.

## 3. Mechanism of SNA Cellular Uptake and Processing in Keratinocytes

**Figure 2:**
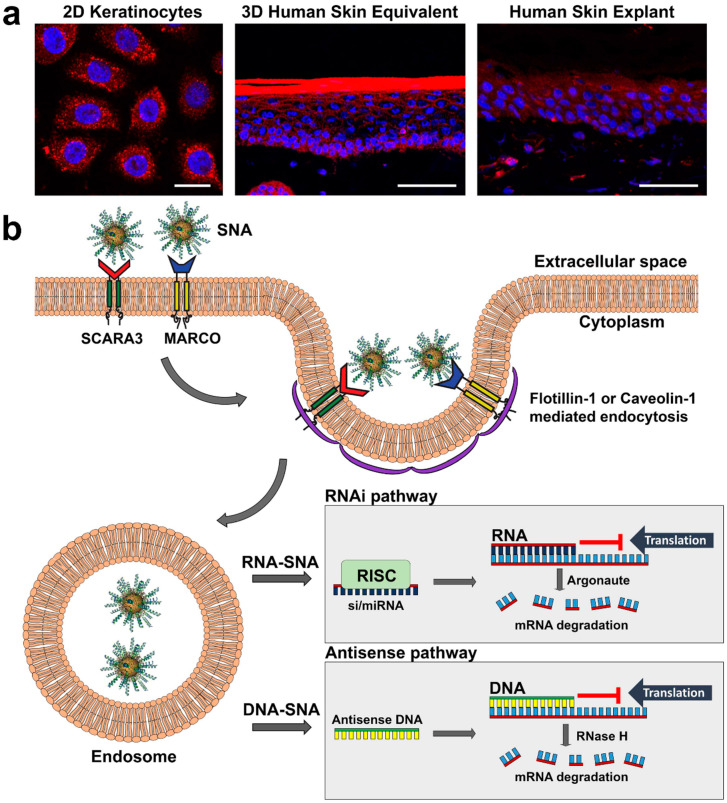
Mechanism of SNA uptake in keratinocytes. (**a**) Confocal imaging of keratinocyte uptake of fluorescently (Cy5) labeled SNAs (red) in 2D, 3D and human explant culture; scale bars in (**a**) from left to right: 20 mm, 50 mm, 50 mm. (**b**) SNAs are: (i) first detected on the keratinocyte cell surface by class A scavenger receptors SCARA3 and MARCO; (ii) taken up primarily by flotillin-1-mediated endocytosis, but also caveolin-1-mediated endocytosis; (iii) deposited into endosomes; and (iv) released from endosomes to suppress gene expression via the RNAi (RNA-SNAs) or antisense (DNA-SNA) pathway; in the RNAi pathway mRNA translation is blocked by antisense RNA or target mRNA is degraded by Argonaute of the RISC complex, whereas in the antisense pathway antisense DNA can block translation or initiate mRNA degradation by RNase H.

Human keratinocytes are known to be difficult to transfect, but SNAs are an effective alternative to standard transfection and transduction techniques. SNAs efficiently penetrate human 3D skin equivalents in vitro [36,59], human explants [59], intact epidermis in Franz cell assays [60], and mouse skin models in vivo [59,60,61,62] (Figure 2a). While the mechanism for penetration into intact epidermis has not been fully elucidated, uptake in keratinocytes has recently been discovered to involve class A scavenger receptors (SR-A) [36,63].

Treatment of keratinocytes and 3D skin equivalents with poly-inosine or fucoidan, which inhibit SR-A function, prevents the uptake of SNAs. However, in contrast with the requirement for scavenger receptor class A member 1 (SCARA1) in endothelial cells [63], uptake into keratinocytes is undertaken predominantly by SCARA3 and, to a lesser extent, macrophage receptor with collagenous structure (MARCO) (Figure 2b). SNAs are internalized predominantly via flotillin-1-mediated endocytosis of scavenger receptor-SNA complexes in differentiated and basal (undifferentiated) keratinocytes, but also via caveolin-1-mediated endocytosis in differentiated keratinocytes (i.e., more towards the epidermal surface) [36].

Once in intracellular endosomes, SNAs are eventually released into the cytoplasm, a process that can be expedited by adding polyethylenimine [64]. After endosomal release SNAs are processed by either the antisense pathway (SNAs with DNA) or the RNAi pathway (SNAs with siRNA or miRNA) (Figure 2b) [53,65]. In the antisense pathway, DNA binds to its mRNA target and either prevents protein translation or attracts RNase H which recognizes DNA bound to mRNA and selectively degrades the mRNA leaving the antisense DNA intact. In the RNAi pathway, siRNA or miRNA from SNAs are cleaved from the surface of the SNA core by Dicer, and the antisense strand is loaded onto the RNA-induced silencing complex (RISC) which guides it to the target mRNA [66,67]. siRNA-induced RNAi leads to cleavage of the mRNA by Argonaute of the RISC complex, whereas miRNA-induced RNAi either prevents translation or initiates mRNA deadenylation thus destabilizing it [66,68,69] (Figure 2b). Though exocytosis of SNAs from cells is known to occur [70], the exact mechanisms are poorly understood.

## 4. Utility and Safety of SNAs

### 4.1. Advantages of SNAs

SNAs have several advantages over other nucleic acid structures. First, SNAs are significantly more stable than linear DNA/RNA of the same sequence, both in aqueous solutions and in vivo, due to their high salt content and the density of oligonucleotides on the nanoparticle core surface [71,72]. SNAs also display a sharper melting transition (2–8 °C vs. ~20 °C) and a higher melting temperature than free oligonucleotides (~43 °C vs. ~37 °C), which in turn has allowed for the use of SNAs as molecular probes [39,65,73]. Both the dense oligonucleotide clustering and high salt content on the surface of SNAs are thought to be responsible for their increased melting temperature, while the high salt concentration has been shown to produce the narrower melting transition [74,75]. Oligonucleotide-containing SNAs also induce a significantly lower immune response compared to normal transfection methods as both RNA and DNA SNAs induced less interferon (IFN) γ release from cells compared to lipofectamine transfection [76]. The most important advantage that SNAs have over free nucleotide strands is an enhanced ability to be taken up by cells without the use of transfection kits or any supplementary reagents [15]. Recent work has also highlighted the ability of SNAs to cross the blood–brain barrier [77,78], which is the basis for a phase 1 clinical trial using intravenously administered Au-NP SNAs to treat glioblastoma (NCT03020017). Despite the robust numbers of SNAs that can enter a single cell, the elicited immune response is minimal and lower than the response using standard transfection reagents [76]. These characteristics make SNAs ideal candidates for use in cutaneous research, given their minimal triggering of immune reactivity, despite the innate immune function of epidermal cells.

### 4.2. Safety of SNAs In Vitro and In Vivo

In primary human keratinocytes Au-NP SNA uptake is dose and time-dependent and has no toxic effects on keratinocytes after at least 4 days of treatment [36,70]. Virtually no cytotoxicity has been seen, even with high SNA concentrations, whereas standard transfection reagents displayed devastating cytotoxicity at comparable siRNA concentrations [31]. In other investigations, Ames texting showed no evidence of mutagenicity, and clastogenicity was not demonstrated in mammalian cell micronucleus testing.

In vivo, Cy5-labeled Au-NP SNAs applied once topically were persistent in mouse skin for up to 10 days, suggesting a depot effect and stability in vivo [60]. Gold-cored SNAs at 0.5 μM (based on gold) were administered daily for 10 days to shaved C57BL/6 mice or for 4 weeks to hairless mice without causing skin inflammation, ulceration, scaling, or color alteration. No significant increase in inflammatory or immune markers in skin (TNFα, IL-6, IFN α and β, or CCL10) were detected in mouse skin treated topically with Au-NP SNAs three times weekly for 3 weeks [60]. No histological abnormalities have been noted in viscera, and the gold content is almost undetectable (0.0003% of the applied dose to liver, 0.00015% in spleen, and undetectable elsewhere). In other preclinical studies, (a) topical applications to 10% of the body surface area of minipigs for 28 days led to no clinical, histological, or necropsy evidence of toxicity (and only 0.025% and 0.01% of the applied dose was detected in the kidneys and liver, respectively); (b) 10 mg/kg topical application to the skin of cynomolgus monkeys caused no toxicity; and (c) repeated application to the mouse ear caused no evidence of lymph node enlargement or inflammation (signs of skin sensitization). Subcutaneous injection for 28 days in rats led to a NOAEL of 2 mg/kg, with minor injection site inflammation that soon subsided, and intravenous injection into monkeys of 2 mg/kg intravenously caused no toxicity. These preclinical safety outcomes have enabled advancement of SNAs towards clinical trials.

In human clinical trials, no toxicity or adverse events were noted when using topically applied L-SNAs (AST-005/XCUR17) to treat psoriasis, and the treatments were well tolerated by all subjects. Clinical trials using subcutaneous injection of L-SNAs (toll-like receptor 9 agonist, cavrotolimod (formerly AST-008)) to stimulate immune responses against skin cancer led to signs of systemic immune activation in the minority of patients, which were dose-related. Specifically, these flu-like symptoms of moderate severity included fatigue (40% of patients), chills (30%), and nausea (30%). In addition, injection site inflammation and/or itch (55% and 20% of patients, respectively), as well as flushed skin (20%) and muscle pain (20%), were observed. A majority (>60%) of these events were associated with the highest dose (32 mg) of AST-008 administered. In addition, presyncope and lymphopenia were each reported in two separate patients, but both subsided spontaneously. No major adverse events or grade 4 toxicity were observed with subcutaneous injection of AST-008. These systemic reactions were not surprising, given the hope that the AST-008 would activate immune responses to target cancer cells; however, more research will be required to optimize the immune-mediated anti-cancer effects of AST-008 after local subcutaneous injection while minimizing potential toxicity. Additionally, more work will be needed to properly characterize the long-term safety of SNAs as a therapeutic option, which will require longer pre-clinical/clinical trials.

## 5. Using SNAs for the Detection and Treatment of Skin Disease

Topical therapeutics for the treatment of skin disease are the gold standard for optimizing effective delivery to the skin and minimizing the risk of systemic side effects. Topical agents have largely been limited to small molecule inhibitors or stimulators, as nucleic acids cannot traditionally penetrate the epidermal barrier. However, SNAs can traverse the stratum corneum and stratum granulosum, sites of epidermal barrier function, and are detectable in the dermis [60], providing a unique opportunity to modify gene expression directly in skin towards more effective treatment and prevention of skin disease. In early studies as proof-of-principle, mouse skin was treated with *EGFR*-targeting Au-NP SNAs for 3 weeks, which resulted in decreased skin thickness and suppressed EGFR signaling [60]. SNAs can also be injected into skin lesions, given their easy accessibility. Table 1 shows preclinical studies and clinical trials of SNAs for skin disorders.

### 5.1. Diabetic Wound Healing

Impaired wound healing is a common complication in patients with type 2 diabetes (T2D). Nearly 10% of all Americans are diagnosed with T2D or pre-T2D, and 25% of these individuals will develop a foot ulcer in their lifetime. However, few treatment options are available. The monosialic ganglioside GM3, located on the outer leaflet of the plasma membrane and the most prevalent sialylated glycosphingolipid in skin, and its synthesizing enzyme, GM3 synthase (GM3S), are critical regulators of insulin resistance in T2D [62,79,80]. Additionally, skin-specific *GM3S* knockout mice fed a high fat diet to induce a T2D state are resistant to impaired wound healing [62]. Thus, GM3S represents a prime target for the treatment of T2D impaired wound healing. 

Using Au-NP siRNA-conjugated SNAs, Randeria et al. successfully targeted *GM3S* expression in 2D keratinocytes and diabetic mouse skin [31] (Table 1). In diabetic mice, *GM3S*-targeted SNAs significantly improved wound healing (as determined by epidermal gap closure), abetted granulation tissue formation, and increased insulin-like growth factor 1 receptor and EGFR signaling [31].

### 5.2. Nanoflares to Detect Abnormal Scar Formation

Most research using SNAs in skin focuses on epidermal and keratinocyte structure and function. However, one recent study investigated the use of specialized SNAs (nanoflares) to identify abnormal gene expression in cutaneous dermal fibroblasts seen in aberrant fibrotic scars [81]. As previously described, nanoflares are Au-NP SNAs functionalized with fluorescently labeled oligonucleotides that only produce a signal upon hybridization to their target mRNA [39].

Hypertrophic and keloid scars are the result of an abnormal fibrotic response to wound healing and are associated with excessive collagen production from increased expression of connective tissue growth factor (CTGF) and transforming growth factor β. Early identification of these scar types is critical for therapeutic intervention, and non-invasive techniques would be ideal for detection, given the role of trauma in causing these abnormal scars. Yeo and colleagues developed *CTGF*-recognizing nanoflares in hopes of identifying fibroblasts in wounds that could induce abnormal scar formation [81]. In 2D primary culture of normal, hypertrophic, and keloidal fibroblasts, *CTGF* nanoflares accurately labelled disease fibroblasts that were overexpressing *CTGF* [81]. Live in vivo imaging detected significantly more nanoflares in mice injected with hypertrophic fibroblasts (HSFs) and *CTGF* nanoflares compared to those injected with normal fibroblasts (NDFs) and *CTGF* nanoflares [81]. Topical application of *CTGF* nanoflares was also successful at identifying hypertrophic fibroblasts in ex vivo human skin injected with NDFs or HSFs. Finally, *CTGF* nanoflares were able to distinguish scarred rabbit ears (post-wounding) from normal, unwounded rabbit ear skin after 48 h of topical application [81]. This research highlights how SNAs can be adapted for both gene regulation and detecting gene expression. Given the ability of SNAs to penetrate skin, this technology could be further adapted for noninvasive detection after topical application to human skin.

### 5.3. Psoriasis

Psoriasis is a common inflammatory skin disorder that is known to result from skewed immune activation towards the Th17/interleukin (IL)-23 and TNFα signaling pathways. While the treatment for patients with moderate-to-severe disease has been revolutionized in both efficacy and safety by the commercial availability of monoclonal antibodies targeting these pathways, the inability of these large monoclonal antibodies to traverse the epidermal barrier has prevented transition of this targeted approach to the 80% of patients with mild-to-moderate psoriasis.

Indeed, using liposomal SNAs (L-SNAs) with DNA targeting *TNFA* (AST-005, generated by Exicure, Inc., Chicago, IL 60614, USA), Lewandowski et al. showed uptake of Cy-5 fluorescent L-SNAs into the markedly thickened human psoriatic skin explants [61]. Using a 3D human raft model of psoriasis, generated by the addition of a cytokine cocktail (TNFα, IL-17 and IL-22), addition of AST-005 to the surface of the raft (simulating topical delivery) led to improvement in the differentiation abnormality of the psoriatic rafts and normalization in the mRNA expression of a variety of psoriatic markers (increases in *TNFA*, *DEFB4*, and *S100A7*, with decreases in *KRT10* and *LOR*, all *p* < 0.001). *TNFA* LSNAs, applied topically every other day in a common moisturizer without penetration enhancers, reversed the development of psoriasis-like disease in the widely used imiquimod-induced mouse model [61]. After just a week of treatment mice showed improvement vs. vehicle and scrambled controls in modified Psoriasis Area and Severity Index scores (epidermal thickness and proliferation, immune cell infiltration, and psoriatic expression markers (all *p* < 0.001)) that was indistinguishable from mice without the psoriatic disease [61].

More recently, an L-SNA targeting *IL17RA* was tested in normal human skin explants, as well as in human 3D and imiquimod-induced mouse models of psoriasis [59]. The L-SNA knocked down IL17RA protein by 66% and 72% in the human normal explant and psoriatic 3D raft models, respectively. In these rafts, expression of psoriatic markers of Th17 pathway activation were also significantly reduced (*IL17C* by 85%, *DEFB4* by 84%, *TNFA* by 77%, and *PI3* by 65%) in comparison with scrambled and vehicle controls (all *p* < 0.001). An L-SNA targeting *IL17RA* in the imiquimod-induced mouse model reduced the modified Psoriasis Area and Severity Index by 74% vs. vehicle-treated mice, reduced epidermal thickness by 56%, and, as in the 3D models, decreased mRNA expression of the various psoriasis markers [59].

These results demonstrated the efficacy of SNAs in penetrating even thickened skin, knocking down gene targets, and reversing skin disease in mouse and human 3D culture models of psoriasis. Studies are ongoing with bispecific L-SNAs that target both TNFα and IL17RA.

### 5.4. Clinical Trials Using SNAs to Treat Skin Pathologies

Owing to their success in ameliorating skin pathologies such as psoriasis and altered wound healing at the pre-clinical level, SNAs are now being developed for use in humans (Table 1). Phase 1 clinical trials have shown good safety and promising results of SNAs as a topical agent directed against TNFα and IL17RA, targets that are suppressed by subcutaneously administered monoclonal antibodies for moderate-to-severe psoriasis (i.e., etanercept, adalimumab, and certolizumab for TNFα and brodalumab for the IL-17 receptor). The first phase 1 study was conducted using L-SNAs (AST-005) to target *TNFA* [82]. Using a template overlying psoriatic lesions (microplaque study), 15 patients were each administered three strengths of AST-005, as well as positive (calcitrotriol cream) and negative (vehicle) controls for 28 days. *TNFA* mRNA expression was decreased with 1% gel vs. vehicle (35% knockdown; *p* = 0.02), with dose responsiveness and no treatment-related adverse events [82]. A subsequent phase 1, similarly designed 25-day clinical trial was conducted using an *IL17RA*-targeting SNA, XCUR17, to treat plaque psoriasis in 21 patients [83]. Prior to the clinical trial, concentrations of XCUR17 applied topically to healthy human skin explants led to knockdown of *IL17RA* mRNA expression of approximately 70% vs. vehicle controls (*p* < 0.001). In the Phase 1 study, no *IL17RA* knockdown was observed in the XCUR17-treated areas versus baseline controls, but significant mRNA reduction in expression of *KRT16*, a marker of psoriasis and hyperproliferation, and a variety of inflammatory markers of psoriasis were also reduced (*IL36A*, *DEFB4*, *IL19*) in XCUR17 vs. vehicle-treated areas. No adverse events were seen [83]. Plans for moving XCUR17 towards Phase 2 trials are in progress.

SNAs can carry immunomodulatory oligonucleotides or antigens, recognized by toll-like receptors, leading to stimulation or regulation of macrophages and antigen-presenting cells [84,85,86]. Cavrotolimod (formerly AST-008) is an L-SNA toll-like receptor 9 (TLR9) agonist that is being used to treat various skin cancers by intratumoral injection. Cavrotolimod activates tumor-based immune responses to encourage tumor cell clearance. In a Phase 1 trial, cavrotolimod was injected subcutaneously in healthy patients to determine its safety and efficacy (NCT03086278) [87]. No serious adverse events or toxicity were reported with injection and a significant increase in lymphocyte infiltration and type 1 immune response was reported [87]. Overall, minor adverse events included flu-like symptoms and injection site reactions. In a Phase 1b open-label trial for advanced or metastatic cutaneous melanoma, Merkel cell carcinoma (MCC), and cutaneous squamous cell carcinoma (cSCC), cavrotolimod was administered in increasing doses (2 to 32 mg) weekly, with the addition (at week 3) of one dose of the FDA-approved programmed cell death protein 1 (PD-1) neutralizing antibody, pembrolizumab [88], which reduces cancer cell immune evasion. Results showed a trend towards increased tumoral immune activity and increased peripheral blood cytokine/chemokines levels in a cavrotolimod dose-dependent manner. Of four subjects with MCC, a difficult cancer to treat, two patients had sustained reduction in tumor size 12 to 24 weeks after starting therapy [89]. A randomized phase 2 clinical trial is currently recruiting and will compare cavrotolimod alone to cavrotolimod plus pembrolizumab (for patients with advanced MCC) or plus the anti-PD-1/PD-L1 medication, cemiplimab (for patients with advanced cSCC) in patients who are recalcitrant to the PD-1 and PD-1/PD-L1 inhibitors alone (NCT03684785) [88,90].

## 6. Conclusions

The impact and potential use of SNAs in both research and clinical applications has helped transform the mechanisms of gene regulation. However, some challenges do exist. Serum proteins are attracted to the oligonucleotide shell of SNAs forming a protein corona [91]. This is especially true of glycine-rich sequences, in which the corona has been shown to increase the rate of macrophage-mediated SNA removal and accumulation in the liver and spleen in mice, thus preventing their uptake by target cells [91,92]. Coating SNAs with PEG to prevent protein corona formation on SNAs has proven beneficial, but it can also reduce cellular uptake [33]. More work will be needed to engineer SNAs to both elude macrophage clearance and maintain a high level of cellular uptake. Once inside the cell, the mechanisms of SNA endosomal/lysosomal release, and ways to maximize this release, will require more attention. Chemical additions on SNAs to improve intracellular trafficking have been developed, but these added chemicals increase the risk of toxicity. Despite progress in achieving SNA cell specificity, the ability of SNAs to be taken up by many cell types handicaps their ability to be cell-type specific with systemic delivery without off-target effects, emphasizing their value when topically applied or injected for skin disease. Lastly, although SNAs have little to no short-term toxicity, the potential long-term toxicity of SNAs, specifically with metal cores, is poorly understood. Coreless or more natural (i.e., liposomal) SNAs may address this concern, but more work is needed.

Despite these challenges, SNAs have made substantial progress both in basic research and clinical use. This is especially true in the field of dermatology because skin offers a unique opportunity to side-step some of the challenges SNAs pose. For example, skin pathologies, being that they are on the skin’s surface, are more easily accessible to drugs. For this reason, using SNAs directly on the skin largely removes the possibility of systemic toxicity or off-target effects. Along the same lines, SNAs topically applied to the skin avoid using the bloodstream, where they are more prone to immune clearance or degradation, to reach their target. Knowing this, combined with the fact that SNAs can penetrate the skin without the use of secondary reagents, SNA use for gene modulation in dermatology is a seemingly perfect match. Continued development of SNAs can expand the use of gene modulation techniques for the topical intervention of cutaneous disease.

## Figures and Tables

**Table 1:** Preclinical and Clinical Trials using Spherical Nucleic Acids in Skin.

Clinical Phase; Status	Skin Disease	Target (Treatment, Dose)	Administration (Sample Size)	Primary Outcome Measure	Secondary Outcome Measures	Results/Outcomes
Pre-clinical (human 3D, mouse); Completed	Psoriasis	TNFα (L-SNA; 50 mM)	Topical, every other day for 1 week (*n* = 12 per group)	Psoriasis severity	Psoriatic marker expression, proliferation	Decreased psoriatic severity, epidermal thickness, immune infiltration, normalization of psoriatic mRNA markers
Pre-clinical (human 3D, mouse); Completed	Psoriasis	IL-17RA (L-SNA; 50 mM)	Topical, daily for 1 week (*n* ≥ 6 per group)	Psoriasis severity	Psoriatic marker expression, proliferation	Decreased psoriatic severity, epidermal thickness and immune infiltration, normalization of psoriatic mRNA markers
Pre-clinical; Completed	Impaired wound healing	GM3S (Au-NP-SNA; 50 nM)	Topical, every other day (*n* = 8 per group)	Wound closure	Granulation tissue, metabolic signaling	Increased wound healing, granulation tissue and IGF1R/EGFR signaling
Phase 1; Completed	Psoriasis	TNFα (AST-005) L-SNA; each subject received vehicle, 0.1%, 0.3%, and 1%	Topical, daily for 28 days (*n* = 15)	Adverse events	*TNFA* knockdown, safety, tolerability, dosing	Well tolerated, no adverse events, significant *TNFA* knockdown
Phase 1; Completed	Psoriasis	IL-17RA (XCUR17) L-SNA; dosage information not available	Topical, daily for 25 days (*n* = 21)	Adverse events	*IL17RA* knockdown, safety, tolerability, dosing, skin inflammation, psoriatic gene expression	Well tolerated, no adverse events, no *IL17RA* knockdown, decreased expression of K16 and inflammatory genes
Phase 1; Completed	Healthy subjects	Toll-like receptor 9 (TLR9) agonist (AST-008) L-SNA: 2–32 mg	Subcutaneous injection, weekly for 9 weeks, then every 3 weeks (*n* = 16)	Adverse events	Recommended dosage, immune response, cytokine/chemokine levels	No serious adverse events: minor injection site reactions and flu-like symptoms reported; increased cytokine/chemokine and immune responses
Phase 1b; Completed	Primarily advanced melanoma, Merkel cell carcinoma (MCC), cutaneous squamous cell carcinoma (cSCC)	TLR9 agonist (AST-008) L-SNA; 2–32 mg with anti-PD-1 antibody (pembrolizumab)	Subcutaneous injection, weekly for 9 weeks then once every 3 weeks (*n* = 20)	Dose escalation study (2–32 mg): adverse events in combination with pembrolizumab	Recommended dosage, immune response	No serious adverse events; dose-related injection site reactions and flu-like symptoms, esp. at 32 mg; dose-related systemic immune activation; more diverse tumoral cellular infiltrate vs. non-injected tumor; increased tumoral cell infiltrate with addition of one dose pembrolizumab
Phase 2; Recruiting	MCC, cSCC	TLR9 agonist (AST-008) L-SNA; 32 mg alone or with anti-PD-1 (MCC; pembrolizumab) or anti-PD-L1 (cSCC; cemiplimab) antibody	Subcutaneous injection, weekly for 9 weeks then once every 3 weeks	Adverse events in combination with pembrolizumab or cemiplimab	Immune and cytokine/chemokine response, tumor size, disease-free survival	Not available; trial ongoing

AST-008: TLR9 agonist L-SNA, now called cavrotolimod; cSCC: Cutaneous squamous cell carcinoma; EGFR: Epidermal growth factor receptor; IGF1R: Insulin-like growth factor-1 receptor; IL-17RA: Interleukin 17 receptor A; L-SNA: Liposomal spherical nucleic acid; MCC: Merkel cell carcinoma; PD-1: Programmed cell death protein-1; TLR9: Toll-like receptor 9, TNFα: Tumor necrosis factor-alpha. An Au-NP SNA targeting Bcl2L12 is currently in a phase 1 clinical trial for glioblastoma (NCT03020017).
