# Correction: Holmes, T.R.; Paller, A.S. Gene Regulation Using Spherical Nucleic Acids to Treat Skin Disorders. *Pharmaceuticals* 2020, *13*, 360

**DOI:** 10.3390/ph18081091

**Published:** 2025-07-23

**Authors:** Thomas R. Holmes, Amy S. Paller

**Affiliations:** Department of Dermatology, Northwestern University Feinberg School of Medicine, Chicago, IL 60611, USA; thomas.holmes@northwestern.edu

**Update to Table**

In Table 1 of the original publication [1], a Result/Outcomes related to preclinical research was never published; as a result, we have now deleted that entry. In addition, at the time of publication, a Phase 2 clinical trial, recruiting at the time, was mentioned; that trial subsequently was terminated and as such this entry row was also deleted with this correction. Also, we decided to delete footnote number 2 since it is not relevant to anything in the table and not relevant to skin disease. The corrected Table 1 appears below. 

**Incorrect Citation and Text Correction**

In the original publication, reference 64 was not the correct citation. The text that the citation was supporting has been updated to better reflect the conclusion and the citation has also been updated to the correct reference, which is reference 18, in the third paragraph of **Section 3. Mechanism of SNA Cellular Uptake and Processing in Keratinocytes**. The first sentence was updated and now states:

“SNAs are eventually released from endosomes into the cytoplasm, a process that can potentially be enhanced by adding polyethylenimine [18].”

**Text Correction**

Unpublished data was included, despite initial acceptance by the peer reviewers. We have removed all unpublished technical data/results. **In Section 4. Utility and Safety of SNAs Sub-Section 4.2. Safety of SNAs In Vitro and In Vivo**, the second paragraph has been updated to say the following:

“In vivo, Cy5-labeled Au-NP SNAs, applied once topically, were persistent in mouse skin for up to 10 days, suggesting a depot effect and stability in vivo [60]. Gold-cored SNAs at 0.5 μM (based on gold) were administered daily for 10 days to shaved C57BL/6 mice or for 4 weeks to hairless mice without causing skin inflammation, ulceration, scaling, or color alteration. No significant increase in inflammatory or immune markers in skin (TNFα, IL-6, IFN α and β, or CCL10) were detected in mouse skin treated topically with Au-NP SNAs three times weekly for 3 weeks [60]. No histological abnormalities were noted in viscera, and the gold content is almost undetectable (0.0003% of the applied dose in liver, 0.00015% in spleen, and undetectable elsewhere). Other preclinical studies have shown low toxicity in pig and monkey models with topical or intravenous application, respectively (Data unpublished; contact authors for more details). These preclinical safety outcomes have enabled advancement of SNAs towards clinical trials.”

Additionally, for the same reasons, unpublished details were removed from **Section 5. Using SNAs for the Detection and Treatment of Skin Disease Sub-Section 5.4. Clinical Trials Using SNAs to Treat Skin Pathologies** in both paragraphs and so the updated paragraphs now read:

“Owing to their success in ameliorating skin pathologies such as psoriasis and altered wound healing at the pre-clinical level, SNAs are now being developed for use in humans (Table 1). Phase 1 clinical trials have shown good safety and promising results of SNAs as a topical agent directed against TNFα and IL17RA, targets that are suppressed by subcutaneously administered monoclonal antibodies for moderate-to-severe psoriasis (i.e., etanercept, adalimumab, and certolizumab for TNFα and brodalumab for the IL-17 receptor). The first phase 1 study was conducted using L-SNAs (AST-005) to target TNFA [81]. A subsequent phase 1, similarly designed 25-day clinical trial was conducted using an IL17RA-targeting SNA (named XCUR17) to treat plaque psoriasis [82].”

This was removed: “Using a template overlying psoriatic lesions (microplaque study), 15 patients were each administered three strengths of AST-005, as well as positive (calcitrotriol cream) and negative (vehicle) controls for 28 days. TNFA mRNA expression was decreased with 1% gel vs. vehicle (35% knockdown; *p* = 0.02), with dose responsiveness and no treatment-related adverse events [81].”

In the next paragraph, we retained:

“SNAs can carry immunomodulatory oligonucleotides or antigens, recognized by toll-like receptors, leading to stimulation or regulation of macrophages and antigen-presenting cells [83–85]. Cavrotolimod (formerly AST-008) is an L-SNA toll-like receptor 9 (TLR9) agonist that is being assessed to treat various skin cancers by intratumoral injection. Cavrotolimod activates tumor-based immune responses to encourage tumor cell clearance. In a Phase 1 trial, cavrotolimod was injected subcutaneously in healthy patients to determine its safety and efficacy (NCT03086278) [86]. No serious adverse events or toxicity were reported with injection and a significant increase in lymphocyte infiltration and type 1 immune response was reported. Overall, minor adverse events included flu-like symptoms and injection site reactions [86]. In a Phase 1b open-label trial for advanced or metastatic cutaneous melanoma, Merkel cell carcinoma (MCC), and cutaneous squamous cell carcinoma (cSCC), cavrotolimod was administered the FDA-approved programmed cell death protein 1 (PD-1) neutralizing antibody, pembrolizumab [87], which reduces cancer cell immune evasion. Results showed a trend towards increased tumoral immune activity and increased peripheral blood cytokine/chemokines levels in a cavrotolimod dose-dependent manner [87].”

This was removed: “Of four subjects with MCC, a difficult cancer to treat, two patients had sustained reduction in tumor size 12 to 24 weeks after starting therapy [89]. A randomized phase 2 clinical trial is currently recruiting and will compare cavrotolimod alone to cavrotolimod plus pembrolizumab (for patients with advanced MCC) or plus the anti-PD-1/PD-L1 medication, cemiplimab (for patients with advanced cSCC) in patients who are recalcitrant to the PD-1 and PD-1/PD-L1 inhibitors alone (NCT03684785) [88,90].”

**References**

Due to the changes above, references 86, and 87 were updated (see below); and references 64, 89 and 90 were removed, which moved all references after 63 up in the order by 1 and the last 2 citations up 3 spots in the order. With this correction, the order of some references has been adjusted accordingly. The authors state that the scientific conclusions are unaffected. This correction was approved by the Academic Editor. The original publication has also been updated.

86. Daniel, W.L.; Lorch, U.; Mix, S.; Bexon, A.S. A first-in-human phase 1 study of cavrotolimod, a TLR9 agonist spherical nucleic acid, in healthy participants: Evidence of immune activation. *Front. Immunol.* **2022**, *13*, 1073777.
87. Milhem, M.M.; Perez, C.A.; Hanna, G.J.; Wise-Draper, T.M.; Bhatia, S.; Bexon, A.S.; Daniel, W.L.; O’Day, S. Phase 1b/2 study of an intratumoral TLR9 agonist spherical nucleic acid (AST-008) and pembrolizumab: Evidence of immune activation. *Cancer Res.* **2020**, *80* (Suppl. 16), LB-140.

The authors state that the scientific conclusions are unaffected. This correction was approved by the Academic Editor. The original publication has also been updated.

## Figures and Tables

**Table 1:** Preclinical and Clinical Trials using Spherical Nucleic Acids in Skin.

Clinical Phase; Status	Skin Disease	Target (Treatment, Dose)	Administration (Sample Size)	Primary Outcome Measure	Secondary Outcome Measures
Pre-clinical (human 3D, mouse); Completed	Psoriasis	TNFα (L-SNA; 50 μM)	Topical, every other day for 1 week (N = 12 per group)	Psoriasis severity	Psoriatic marker expression, proliferation
Pre-clinical (human 3D, mouse); Completed	Psoriasis	IL-17RA (L-SNA; 50 μM)	Topical, daily for 1 week (N ≥ 6 per group)	Psoriasis severity	Psoriatic marker expression, proliferation
Pre-clinical; Completed	Impaired wound healing	GM3S (Au-NP-SNA; 50 nM)	Topical, every other day (N = 8 per group)	Wound closure	Granulation tissue, metabolic signaling
Phase 1; Completed	Psoriasis	TNFα (AST-005) L-SNA; each subject received vehicle, 0.1%, 0.3%, and 1%	Topical, daily for 28 days (N = 15)	Adverse events	TNFα knockdown, safety, tolerability, dosing
Phase 1; Completed	Psoriasis	IL-17RA (XCUR17) L-SNA; dosage information not available	Topical, daily for 25 days (N = 21)	Adverse events	Il-17RA knockdown, safety, tolerability, dosing, skin inflammation, psoriatic gene expression
Phase 1; Completed	Healthy subjects	Toll-like receptor 9 (TLR9) agonist (AST-008 ^1^) L-SNA; 2–32 mg	Subcutaneous injection, weekly for 9 weeks then every 3 weeks (N = 16)	Adverse events	Recommended dosage, immune response, cytokine/chemokine levels
Phase 1b; Completed	Primarily advanced melanoma,Merkel cell carcinoma (MCC), cutaneous squamous cell carcinoma (cSCC)	TLR9 agonist (AST-008) L-SNA; 2–32 mg with anti-PD-1 antibody (pembrolizumab)	Subcutaneous injection, weekly for 9 weeks then once every 3 weeks (N = 20)	Dose escalation study (2–32 mg): adverse events in combination with pembrolizumab	Recommended dosage, immune response

^1^ AST-008: TLR9 agonist L-SNA, now called cavrotolimod; cSCC: Cutaneous squamous cell carcinoma; EGFR: Epidermal growth factor receptor; IGF1R: Insulin-like growth factor-1 receptor; IL-17RA: Interleukin 17 receptor A; L-SNA: Liposomal spherical nucleic acid; MCC: Merkel cell carcinoma; PD-1: Programmed cell death protein-1; TLR9: Toll-like receptor 9, TNFα: Tumor necrosis factor α.

## References

[1] Holmes T.R., Paller A.S. (2020). Gene Regulation Using Spherical Nucleic Acids to Treat Skin Disorders. Pharmaceuticals.

